# Supplementary material for: Rapid vertical exchange at fronts in the Northern Gulf of Mexico
Source: Nat Commun. 2022 Sep 26;13:5624. doi: 10.1038/s41467-022-33251-7 (PMC9512905; doi:10.1038/s41467-022-33251-7)
Supplement: Supplementary file 1 — Supplementary Information [file 41467_2022_33251_MOESM1_ESM.pdf]

Supplementary Materials for

Rapid Vertical Exchange at Fronts in the Northern Gulf of Mexico

Lixin Qu*, Leif N. Thomas*, Aaron F. Wienkers, Robert D. Hetland, Daijiro Kobashi, John R. Taylor, Fucent Hsuan Wei Hsu, Jennifer A. MacKinnon, R. Kipp Shearman, and Jonathan D. Nash

*Corresponding authors. Email: lixinqu123@gmail.com and leift@stanford.edu

This PDF file includes:

Supplementary Figures: Figs. S1 to S7

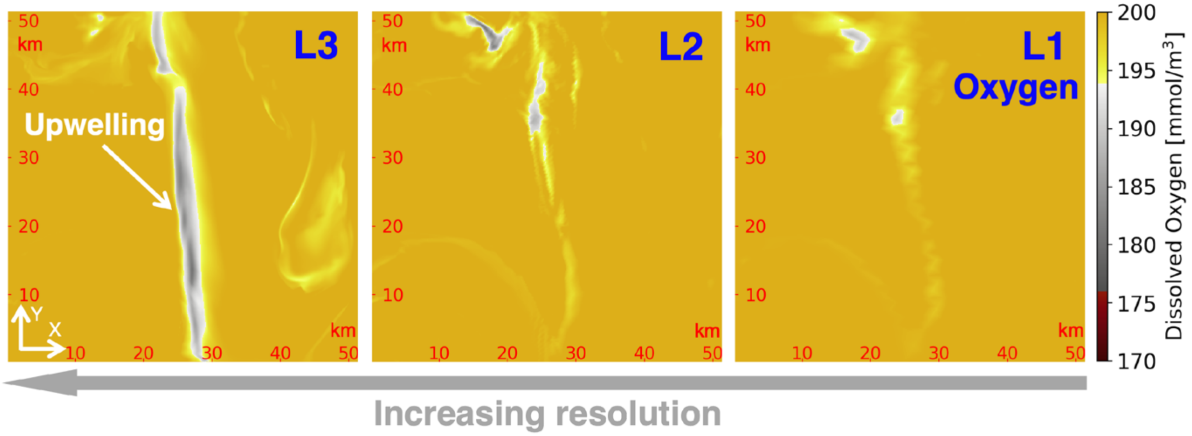

Fig. S1. Horizontal slices of dissolved oxygen. These slices are made over the L3 domain from the L1, L2, and L3 simulations. The horizontal resolutions of L1-3 are O(1 km), O(300 m), and 100 m. The L1 and L2 simulations are interpolated to the L3 grid for comparison. The slices are made at the subsurface (z = -4.7 m). Upwelling of low-oxygen water near the front is most prominent in L3 but less obvious in L1 and L2. The poor representation is due to the relatively low resolutions of L1 and L2 simulations. All the model snapshots are taken on June 15, 2010 00:00 UTC.

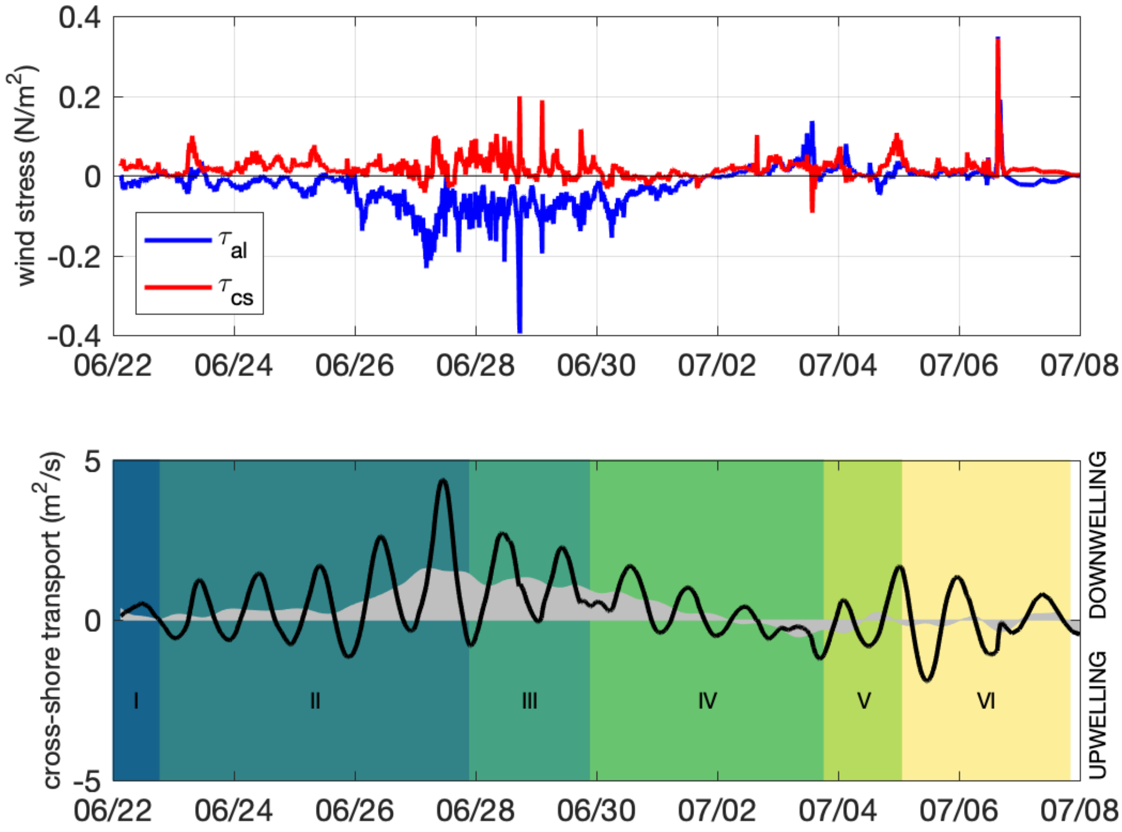

Fig. S2. Overview of the wind-forcing experienced on the cruise and the different flow regimes that were observed. Upper panel: along-shore (blue) and cross-shore (red) components of the wind-stress as inferred from the winds measured on the RV Walton Smith. Lower panel: the cross-shore transport of water (black) as estimated using a slab mixed layer model forced by the observed winds. The low-pass filtered transport is shaded in gray, where a positive (negative) cross-shore transport would drive downwelling (upwelling). The six flow regimes that were experienced are indicated in color: I) well mixed ocean following Tropical Storm Claudette, II) slumping isopycnals and inertial freshwater bore, III) downwelling winds mix ocean, push freshwater onshore, IV) bottom-attached shallow freshwater front and instabilities, V) winds relax and reverse, offshore propagation and detachment of shallow freshwater front, VI) interaction of shallow freshwater front with inertial motions.

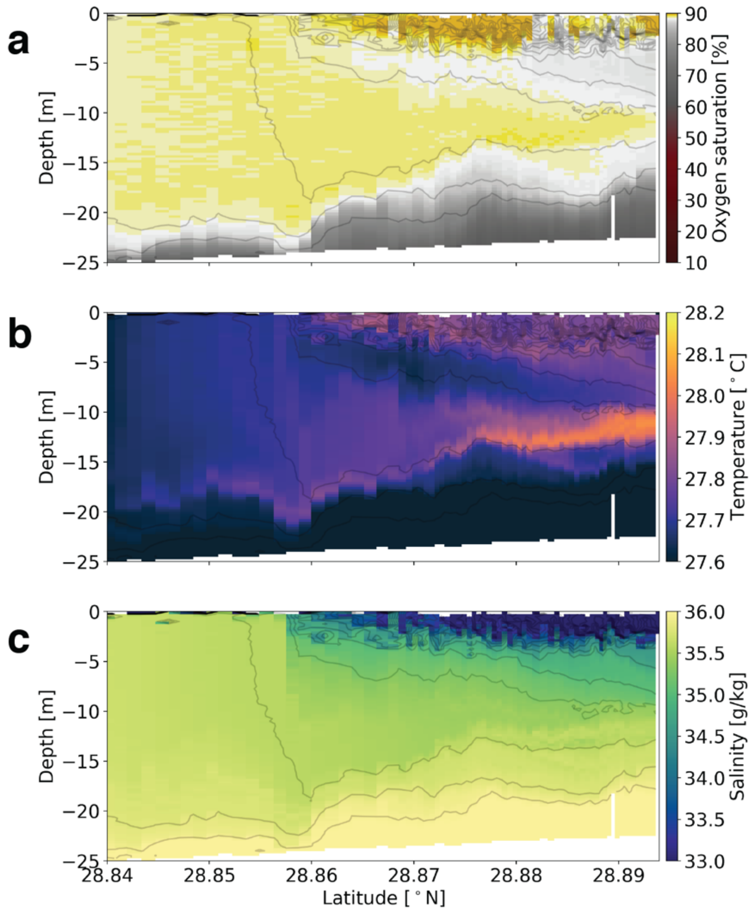

Fig. S3. SUNRISE Campaign transects on June 23, 2021. Vertical sections of oxygen saturation (a), temperature (b), and salinity (c) observed with R/V Pelican between 10:00 and 14:00 UTC on June 23, 2021. To illustrate the frontal processes, the offshore fraction of the transect with the depth deeper than 25 m is not shown. Grey contours are isopycnals drawn every 0.2 kg/m^3. The ship track of R/V Pelican associated with these transects is shown in Fig. 2.

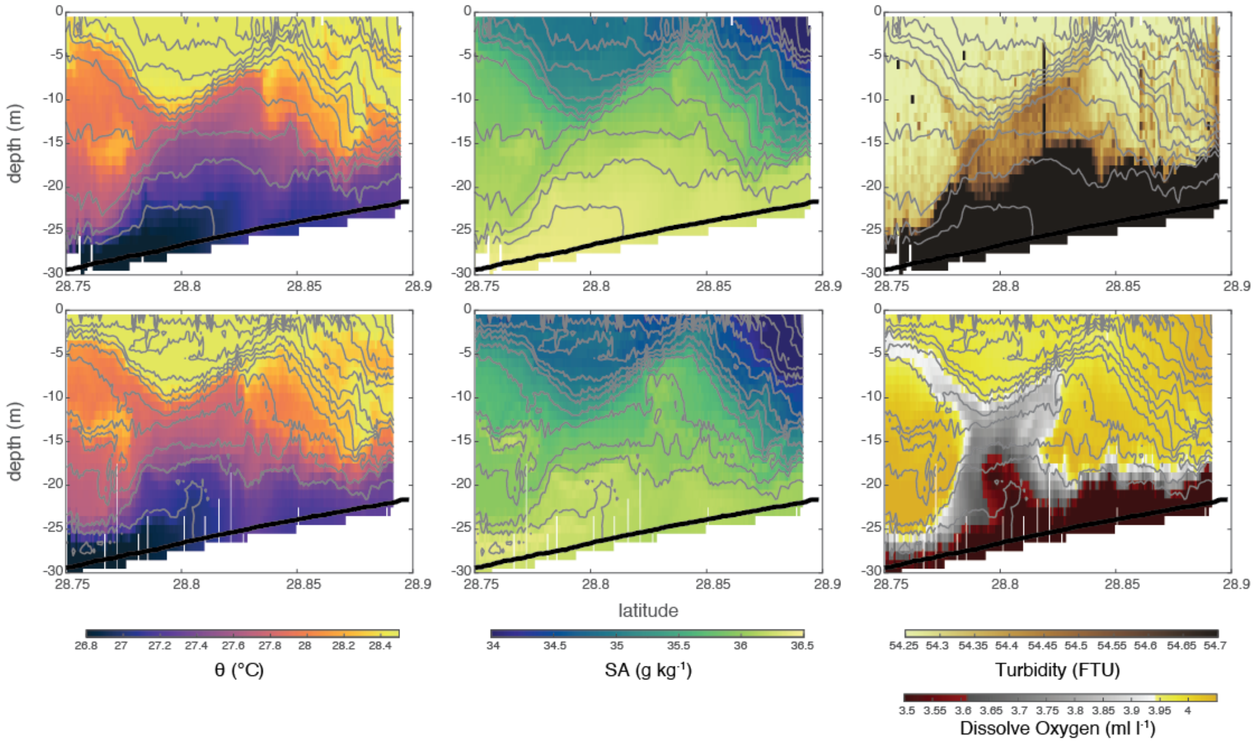

Fig. S4. SUNRISE Campaign transect on June 25, 2021. Vertical sections of temperature (left), salinity (middle), and turbidity/dissolved oxygen (right) observed with R/Vs Pelican (upper) and Walton Smith (lower) between 17:00 and 21:00 UTC on June 25, 2021. Grey contours are isopycnals drawn every 0.2 kg/m^3. Black lines show the bottom located by the ADCPs in the bottom track mode. The ship tracks of R/Vs Walton Smith and Pelican associated with these transects are shown in Fig. 2.

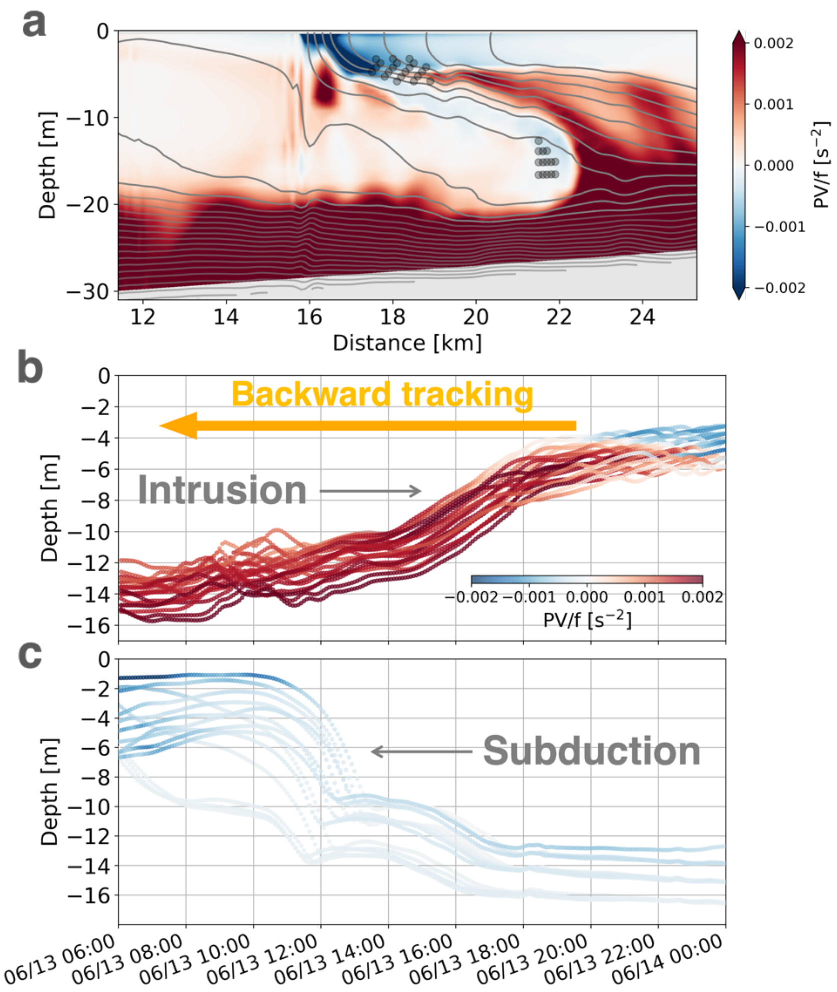

Fig. S5. Lagrangian float tracking. (a) Across-front section of PV. The section is made at June 14, 2010 00:00 UTC along the orange line marked in Fig. 3e. Isopycnals are contoured every 0.2 kg/m^3 in gray. (b) Time series of float depth colored by PV. The floats are initially released at the positions marked by the upper group of dots in (a) and backward tracked in time. (c) Same as b but for the floats marked by the lower dots in (a). The results are made from the TXLA-L3 solution.

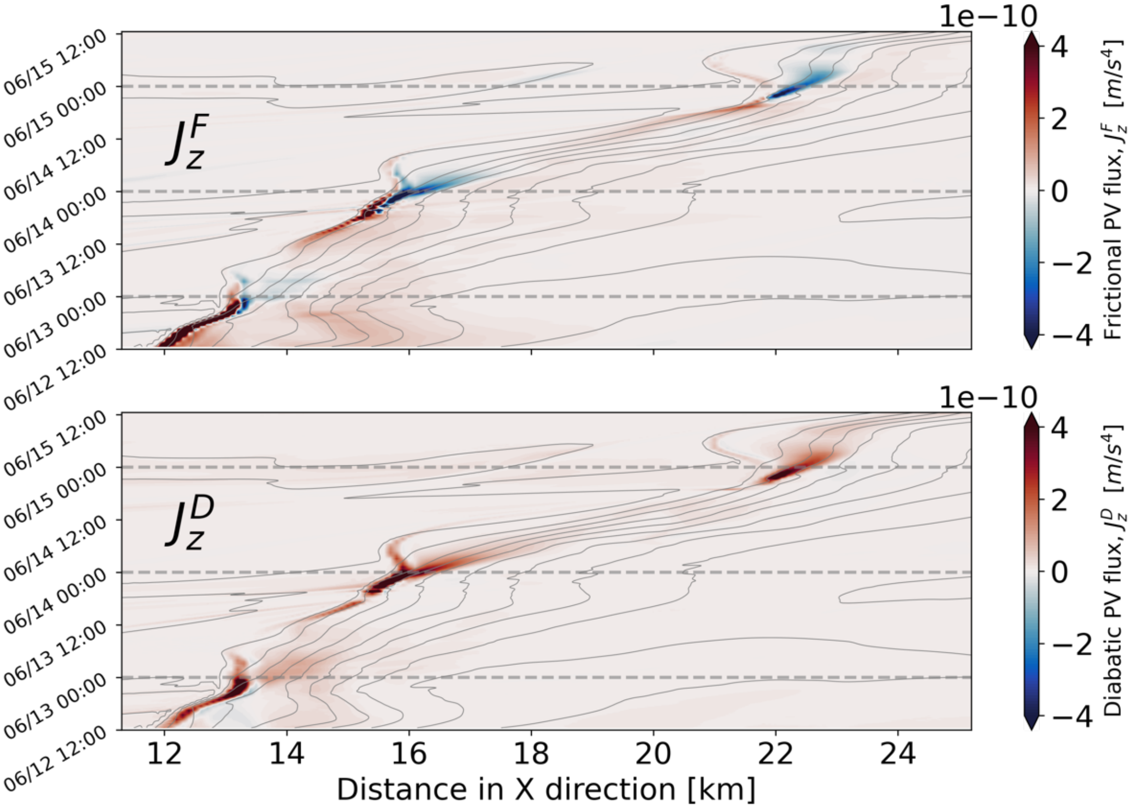

Fig. S6. Hovmöller diagrams of surface PV flux. The frictional PV flux $J_z^F = (\nabla_h b \times \mathbf{F_h}) \cdot \hat{\mathbf{z}}$ and the diabatic PV flux $J_z^D = -(f + \zeta)D$ are shown as a function of distance in the x direction and time (b is the buoyancy, $\mathbf{F_h}$ is the horizontal frictional body force, ζ is the relative vorticity, and D represents the diabatic processes). $\mathbf{F_h}$ and D are obtained using the diagnostics from the TXLA-L3 solution. The diagrams are made along the across-front section marked by the orange line in Fig. 5b. The gray contours are isopycnals made every 0.2 kg/m^3. The time is in UTC. The calculations are made from the TXLA-L3 solution.

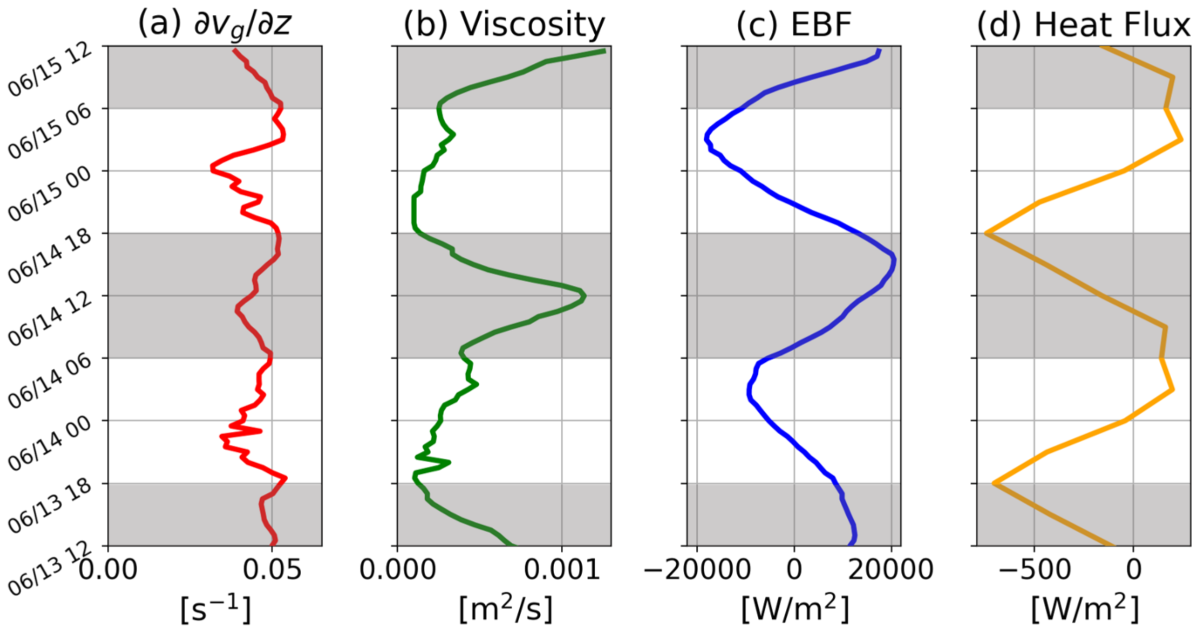

Fig. S7. Evolution of frontal properties on the light side of the front over two inertial periods. (a) Time-series of the geostrophic shear in the y direction at z=-5 m. (b) Time-series of the viscosity at z=-5 m. (c) Time series of the time-dependent EBF. The Ekman transport in the EBF is calculated via multiplying the slab mixed layer model solution for the velocity by the mixed layer thickness H=5m, i.e., $\mathbf{M_e} = (u_{ML}\hat{\mathbf{x}} + v_{ML}\hat{\mathbf{y}})H$. Positive values indicate a tendency of destabilizing the front. (d) Time series of surface heat flux. Positive values mean buoyancy loss. All the properties are averaged over a 2 km-long section at the light side of the front (i.e., the first 2 km of the magenta section in Fig. 5e). The shaded areas mark the periods when the viscosity is enhanced. The time is in UTC. The calculations are made from the TXLA-L3 solution.
