# Supplementary material for: Rapid vertical exchange at fronts in the Northern Gulf of Mexico
Source: Nat Commun. 2022 Sep 26;13:5624. doi: 10.1038/s41467-022-33251-7 (PMC9512905; doi:10.1038/s41467-022-33251-7)
Supplement: Supplementary file 3 — Reporting Summary [file 41467_2022_33251_MOESM3_ESM.pdf]

nature portfolio

Corresponding author(s): Lixin Qu

Last updated by author(s): Aug 1, 2022

Reporting Summary

Nature Portfolio wishes to improve the reproducibility of the work that we publish. This form provides structure for consistency and transparency in reporting. For further information on Nature Portfolio policies, see our Editorial Policies and the Editorial Policy Checklist.

Statistics

For all statistical analyses, confirm that the following items are present in the figure legend, table legend, main text, or Methods section.

n/a	Confirmed	
☒	☐	The exact sample size (*n*) for each experimental group/condition, given as a discrete number and unit of measurement
☒	☐	A statement on whether measurements were taken from distinct samples or whether the same sample was measured repeatedly
☒	☐	The statistical test(s) used AND whether they are one- or two-sided<br>*Only common tests should be described solely by name; describe more complex techniques in the Methods section.*
☒	☐	A description of all covariates tested
☒	☐	A description of any assumptions or corrections, such as tests of normality and adjustment for multiple comparisons
☒	☐	A full description of the statistical parameters including central tendency (e.g. means) or other basic estimates (e.g. regression coefficient) AND variation (e.g. standard deviation) or associated estimates of uncertainty (e.g. confidence intervals)
☒	☐	For null hypothesis testing, the test statistic (e.g. *F*, *t*, *r*) with confidence intervals, effect sizes, degrees of freedom and *P* value noted<br>*Give P values as exact values whenever suitable.*
☒	☐	For Bayesian analysis, information on the choice of priors and Markov chain Monte Carlo settings
☒	☐	For hierarchical and complex designs, identification of the appropriate level for tests and full reporting of outcomes
☒	☐	Estimates of effect sizes (e.g. Cohen's *d*, Pearson's *r*), indicating how they were calculated

Our web collection on statistics for biologists contains articles on many of the points above.

Software and code

Policy information about availability of computer code

Data collection	ROMS and CROCO are employed to conduct the numerical simulations. The source code of ROMS is available at https://www.myroms.org. The source code of CROCO is available at https://www.croco-ocean.org.
Data analysis	The source code of ADCP processing software, UHDAS+CODAS, is available at http://uhdas.org. The cartographic package used for the visualization in this study is Cartopy, and its source code and the associated geospatial data are available at https://github.com/SciTools/cartopy. The package used for the particle tracking is Pyticles, which is available at https://doi.org/10.5281/zenodo.4973786. The code used for the post-processing of simulation output is XROMS, which is archived at https://github.com/hetland/xroms.

For manuscripts utilizing custom algorithms or software that are central to the research but not yet described in published literature, software must be made available to editors and reviewers. We strongly encourage code deposition in a community repository (e.g. GitHub). See the Nature Portfolio guidelines for submitting code & software for further information.

Data

Policy information about availability of data

All manuscripts must include a data availability statement. This statement should provide the following information, where applicable:

- Accession codes, unique identifiers, or web links for publicly available datasets
- A description of any restrictions on data availability
- For clinical datasets or third party data, please ensure that the statement adheres to our policy

The satellite imageries used in this study are from the Suomi-NPP/VIIRS Ocean Color Data Product and available online through the WORLDVIEW portal supported by NASA: https://worldview.earthdata.nasa.gov. The map background is the NASA Blue Marble which is available at https://visibleearth.nasa.gov/collection/1484/blue-marble. The bathymetry dataset used in the TXLA simulation is ETOPO5 which is available at https://www.ngdc.noaa.gov/mgg/global/etopo5.HTML. The simulation forcing datasets, Global HYCOM Reanalysis and ERA-interim, are available at http://www.hycom.org and https://www.ecmwf.int/en/forecasts/datasets/reanalysis-datasets/era-interim, respectively. The river discharge data are obtained from U.S. Army Corps of Engineers (https://www.mvn.usace.army.mil/) and U.S. Geological Survey (https://waterdata.usgs.gov/nwis). The data of the TXLA simulation and the forward/backward particle tracking are available online at https://doi.org/10.5281/zenodo.6381139. The data of the SUNRISE Campaign 2021 used in this study are available online at https://doi.org/10.5281/zenodo.6381027.

Human research participants

Policy information about studies involving human research participants and Sex and Gender in Research.

Reporting on sex and gender	n/a
Population characteristics	n/a
Recruitment	n/a
Ethics oversight	n/a

Note that full information on the approval of the study protocol must also be provided in the manuscript.

Field-specific reporting

Please select the one below that is the best fit for your research. If you are not sure, read the appropriate sections before making your selection.

☐ Life sciences ☐ Behavioural & social sciences ☒ Ecological, evolutionary & environmental sciences

For a reference copy of the document with all sections, see nature.com/documents/nr-reporting-summary-flat.pdf

Ecological, evolutionary & environmental sciences study design

All studies must disclose on these points even when the disclosure is negative.

Study description	Using high-resolution ocean observations and numerical simulations, we demonstrate how the summer land-sea breeze generates rapid vertical exchange at the Mississippi/Atchafalaya River plume fronts in the Northern Gulf of Mexico.
Research sample	The ocean observations and numerical simulations are focused on the Mississippi/Atchafalaya River plume fronts in the Northern Gulf of Mexico. The latitude of the Northern Gulf of Mexico is near 30N so that near-inertial motions are resonantly generated by the diurnal land-sea breeze. At the same time, the submesoscale fronts are ubiquitous in the Mississippi/Atchafalaya River plume. Consequently, the Northern Gulf of Mexico is an ideal, natural geophysical lab to study the interactions between submesoscales and near-inertial motions and the associated vertical transport as demonstrated in this study.
Sampling strategy	Two research vehicles were coordinated to simultaneously sample on repeating, parallel transects. The transects were focused on the Mississippi/Atchafalaya River plume fronts. This sampling strategy with the coordination of two research vehicles ensures the calculations of the gradients of velocity and buoyancy and supports this study as a cornerstone.
Data collection	A oceanic field campaign was launched in the summer of 2021 from June 19th to July 9th. The data were collected by the Master and crew of the R/V Pelican and the Master and crew of R/V Walton Smith. The data collected during the campaign include hydrography (such as temperature and salinity) using the instruments, Vertical Microstructure Profilers, and velocity measurements made using the instruments, 600 kHz (pole-mounted) and 1200 kHz (ship-mounted) Acoustic Doppler Current Profilers. NASA Ocean Color Data Product was used to guide the field campaign. Along with the observational data, the numerical simulations were conducted after the field campaign on the supercomputers of Texas A&M University and Stanford University by the modeling group of SUNRISE (https://sunrise-nsf.github.io/) to study the underlying physics.
Timing and spatial scale	Both the ocean observations and numerical simulations are focused on the Mississippi River plume fronts in the Northern Gulf of

Timing and spatial scale	Mexico during summertime. The summertime in the Northern Gulf is the time when the research objects, i.e., the near-inertial motions and the submesoscale fronts, are assembled in the nature. The field campaign was launched in the summer of 2021 from June 19th to July 9th, and the simulations were in the summer of 2010 from June 1st to July 26th.
Data exclusions	n/a
Reproducibility	n/a
Randomization	n/a
Blinding	n/a

Did the study involve field work? ☒ Yes ☐ No

Field work, collection and transport

Field conditions	The diurnal land-sea breeze was notable, and the near-inertial (diurnal) motions of the plume fronts and the vertical transport were significant.
Location	Northern Gulf of Mexico. 28.6N-29.1N. 91.9W-91.7W.
Access & import/export	n/a
Disturbance	n/a

Reporting for specific materials, systems and methods

We require information from authors about some types of materials, experimental systems and methods used in many studies. Here, indicate whether each material, system or method listed is relevant to your study. If you are not sure if a list item applies to your research, read the appropriate section before selecting a response.

Materials & experimental systems

n/a	Involved in the study	
☒	☐	Antibodies
☒	☐	Eukaryotic cell lines
☒	☐	Palaeontology and archaeology
☒	☐	Animals and other organisms
☒	☐	Clinical data
☒	☐	Dual use research of concern

Methods

n/a	Involved in the study	
☒	☐	ChIP-seq
☒	☐	Flow cytometry
☒	☐	MRI-based neuroimaging
